# Opportunity and accessibility: an environmental scan of publicly available data repositories to address disparities in healthcare decision-making

**DOI:** 10.1186/s12939-024-02187-3

**Published:** 2024-05-08

**Authors:** Lydia Vinals, Amruta Radhakrishnan, Grammati Sarri

**Affiliations:** 1 Cytel Inc, 1 University Avenue, 3rd Floor, Toronto, M5J 2P1 Canada; 2 Cytel Inc, Hamilton House, Mabledon Place, London, WC1H 9BB UK

**Keywords:** Health disparities, Publicly available data sources, Healthcare decision-making, Health technology assessment

## Abstract

**Background:**

Health disparities, starkly exposed and exacerbated by coronavirus disease 2019, pose a significant challenge to healthcare system access and health outcomes. Integrating health inequalities into health technology assessment calls for robust analytical methodologies utilizing disaggregated data to investigate and quantify the scope of these disparities. However, a comprehensive summary of population datasets that can be used for this purpose is lacking. The objective of this review was to identify publicly accessible health inequalities data repositories that are potential resources for healthcare decision-making and future health technology assessment submissions.

**Methods:**

An environmental scan was conducted in June of 2023 of six international organizations (World Health Organization, Organisation for Economic Co-operation and Development, Eurostat, United Nations Inter-agency Group for Child Mortality Estimation, the United Nations Sustainable Development Goals, and World Bank) and 38 Organisation for Economic Co-operation and Development countries. The official websites of 42 jurisdictions, excluding non-English websites and those lacking English translations, were reviewed. Screening and data extraction were performed by two reviewers for each data repository, including health indicators, determinants of health, and health inequality metrics. The results were narratively synthesized.

**Results:**

The search identified only a limited number of country-level health inequalities data repositories. The World Health Organization Health Inequality Data Repository emerged as the most comprehensive source of health inequality data. Some country-level data repositories, such as Canada’s Health Inequality Data Tool and England’s Health Inequality Dashboard, offered rich local insights into determinants of health and numerous health status indicators, including mortality. Data repositories predominantly focused on determinants of health such as age, sex, social deprivation, and geography.

**Conclusion:**

Interactive interfaces featuring data exploration and visualization options across diverse patient populations can serve as valuable tools to address health disparities. The data they provide may help inform complex analytical methodologies that integrate health inequality considerations into healthcare decision-making. This may include assessing the feasibility of transporting health inequality data across borders.

**Supplementary Information:**

The online version contains supplementary material available at 10.1186/s12939-024-02187-3.

## Introduction

The Rio Political Declaration on Social Determinants of Health was ratified in 2011 and was endorsed by many of the World Health Organization (WHO) Member States [1]. This declaration represents a global political commitment to reducing health inequalities from a perspective that recognizes the significant influence of social, economic, and environmental factors on health and well-being. Identifying health inequalities (differences in health across population subgroups) is an integral part of addressing health inequities (differences in health that are deemed unfair or ethically problematic). Toward achieving this aim, the Rio Declaration specifically highlighted the need to create, enhance, and maintain health information systems that provide disaggregated data by dimensions of inequality (broad criteria by which population subgroups are defined) [1]. Such information systems also provide invaluable information for global monitoring of the Sustainable Development Goals (SDG) formulated in 2015 by the United Nations (UN) General Assembly as the 2030 Agenda for Sustainable Development [2].

The 2020 WHO Global Report on Health Data Systems and Capacity evaluated health information systems from 133 countries, representing 87% of the global population [3]. The report emphasized that while 90% of countries published progress reports on their national health sector strategic plan in the last five years, only 56% examined gender-based inequalities and even fewer (38%) explored disparities related to socioeconomic status [3]. Only 51% of countries had disaggregated population projections [3]. The lack of reporting of disaggregated data limits the capacity to establish links between social determinants of health and health outcomes and may obscure health inequalities.

Disaggregated data also allow tracking changes in health inequalities over time to help inform interventions and policy and assess if they are working fairly for everyone. However, a 2020 environmental scan of health equity initiatives from 36 high-income Organisation for Economic Co-operation and Development (OECD) countries found that only seven (19%) had published reports exploring socioeconomic disparities (e.g., income, education, material deprivation) in health over time [4]. Other important determinants of health beyond socioeconomic status, such as gender, were evaluated in a 2022 report from the Pan-Canadian Health Inequalities Reporting Initiative. Of the 19 countries considered, 13 (68%) published reports on changes in health inequalities; sex/gender, education, age, geography, and area-level deprivation were the top 5 reported determinants of health.

To adequately understand and quantify the extent and impact of health inequalities, disaggregated health data should be accessible to decision-makers [3]. While data access and sharing have improved in recent years, disaggregated data on health indicators are still not widely accessible and not shared extensively [3]. Only 40% of countries have a well-developed or higher capacity for data access and sharing, but aggregated data remain the most shared form of data [3]. Furthermore, only 25% of countries update their global health portal more than once a year [3].

Health inequalities have serious implications for underserved and marginalized populations and are thus a crucial contextual factor to consider when evaluating the value of new health technologies. Historically, health inequalities have not been a core component in health technology assessment (HTA) value frameworks and payer interactions [5, 6]. However, HTA bodies are progressively shifting toward recognizing the importance of integrating health inequalities into their evaluation of new health technologies [7–11]. Of note, the WHO recently published a detailed framework outlining key criteria to consider in HTA. This framework included established criteria such as assessing safety and clinical effectiveness, incorporating economic considerations, conducting budget impact analyses, evaluating organizational impact, feasibility considerations, and acceptability to healthcare providers and patients, and noted equity and ethical issues as being highly relevant [12]. The acknowledgement of the significance of equity considerations has led to investing in databases that accurately represent diverse populations, consider access barriers, and thereby promote greater inclusivity [13]. Health inequality considerations in HTA are nascent without clearly defined or consistent methods for their integration in the evaluation of new health technologies. In a recent paper aiming to establish methods for incorporating health equity considerations in the HTA process, the Institute for Clinical and Economic Review (ICER) highlighted the diverse forms of evidence that can be used to quantify and assess health inequalities, ranging from qualitative to quantitative measures like equity-informed economic evaluations [8]. Even without quantitative measures, ICER stressed the importance of incorporating epidemiological data on the prevalence of a given disease in different subpopulations as well as disparities in access to treatment and outcomes [8]. Undoubtedly, integrating health inequality considerations in HTA necessitates equity-relevant data.

Broadening health inequality monitoring through accessible, good-quality disaggregated data can help uncover the links between determinants of health and health outcomes by facilitating the tracking of health equality goals and the assessment of the impact of new interventions on existing health inequalities. To support researchers and healthcare decision-makers in addressing health disparities across the globe, it is essential to have an up-to-date overview of publicly available data repositories on a population-level that provide disaggregated data on health indicators and determinants of health. An environmental scan was conducted to identify and summarize key elements from national and global, freely accessible data repositories, aiming to provide a clear resource for those concerned with understanding the extent of health inequalities and how they may be considered in healthcare decision-making.

## Methods

An environmental scan was performed by two reviewers (LV, AR) to identify freely accessible, web-based health inequalities data repositories. A pre-defined protocol was developed to guide this scanning. The focus was on identifying data repositories reporting disaggregated data categorized by determinants of health (e.g., sex, age, urbanization, geography, disability, economic status, education) and health indicators (e.g., morbidity, mortality). Indicators beyond health, such as those pertaining to the SDGs (e.g., environmental degradation, women’s empowerment index, development indices, multidimensional poverty index, and child protection indicators), were beyond the scope of this review.

HTA institutions utilize rigorous and systematic methods to guide decision-making. Given its resource-intensive nature, established HTA processes tend to be less common in low- and-middle income countries which may lack sufficient data, and requisite decision-making systems [14]. Therefore, this review focused on six international organizations (WHO, OECD, Eurostat, the Inter-agency Group for Child Mortality Estimation [IGME], the World Bank, and the UN SDG) and the 38 OECD member countries to highlight upper-middle- to high-income countries (i.e., the majority of which have established healthcare information systems and resources for monitoring populations and health risks [3]). For the United Kingdom, individual searches were conducted for England, Scotland, Wales, and Northern Ireland, given the devolution of health services. In total, 42 jurisdictions and six international organizations were reviewed using their official websites. Data repositories available in English or available on an online platform allowing web-based translation into English were included.

Two reviewers independently reviewed the official websites of included countries and international organizations. For countries or international organizations with publicly available web-based health inequalities data repositories, availability of the following elements was extracted: health indicators (outcomes), determinants of health (exposures), health inequality metrics, data visualization features, and downloadable formats. To enable comparisons between identified data repositories, available health indicators (outcomes) and determinants of health (exposures) were grouped thematically by definition.

## Results

Of the 42 countries and six international organizations reviewed, 14 (29%) had publicly available web-based health inequalities data repositories. All international organizations reviewed had publicly available, global, web-based health inequalities data repositories, including the WHO (Health Inequality Data Repository) [15], the OECD (OECD.Stat) [16], the UN SDG (SDG Global Database) [17], the World Bank (DataBank) [18], IGME (UN Inter-agency Group for Child Mortality Estimation) [19], and Eurostat (Eurostat) [20].

The WHO Health Inequality Data Repository [15], which was launched in 2023, was the most extensive compilation of publicly accessible disaggregated data, comprising more than 2,000 health indicators and 22 determinants of health. It draws upon 59 datasets, including those provided by the OECD, UN SDG, World Bank, IGME, and Eurostat. However, several criteria were employed in selecting data from these international sources for inclusion in the WHO Health Inequality Data Repository and, consequently, not all available data from these international organizations may be accessible through the WHO Health Inequality Data Repository. As a result, the five other publicly accessible international organizations were separately reviewed and treated as independent, web-based repositories for health inequality data.

Eight of the 42 countries reviewed had web-based health inequalities data repositories. These included Australia (Australian Health Performance Framework [AHPF] [21]), Belgium (Belgian Health Interview Survey – Interactive Analysis [HISIA] [22]), Canada (Health Inequalities Data Tool) [23], England (Health Inequalities Dashboard [24], the Segment Tool [25], and the COVID-19 Health Inequalities Monitoring for England [CHIME] tool [26]), Finland (StatFin) [27], Norway (Norhealth) [28], the United States (US) (Interactive Atlas of Heart Disease and Stroke [29]; United States Diabetes Surveillance System [30]), and Wales (Public Health Wales Observatory [31]). England had three separate web-based health inequalities data repositories. The Health Inequalities Dashboard offered information on health indicators broken down by various determinants of health. The Segment Tool provided data concerning the causes of death and age groups that contribute to disparities in life expectancy. Additionally, the CHIME tool presented data specifically related to coronavirus disease 2019 (COVID-19). The US had two disease-specific, web-based health inequality repositories: one for heart disease and stroke and the other for diabetes.

**Fig. 1:**
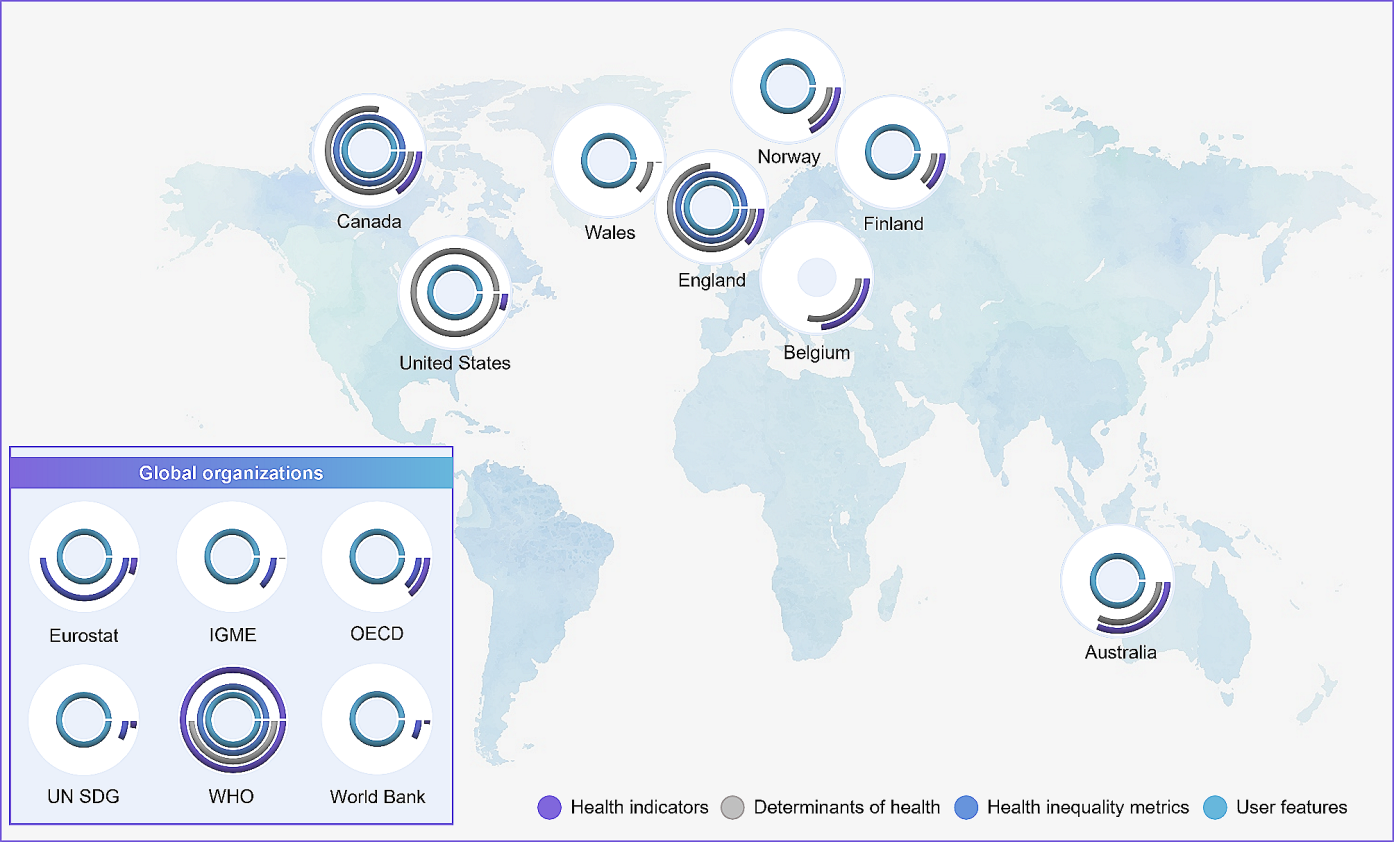
*Characteristics of Identified Web-based Health Inequalities Data Repositories.* The gauge charts represent the availability of health indicators, determinants of health, health inequality metrics, and user features (data visualization and downloadable exports) across identified data repositories. For health indicators and determinants of health, the colored segments represent the percentage of health indicators and determinants of health available in each data repository as a proportion of the highest number reported across all identified data repositories. Health inequality metrics and user features were treated as binary characteristics (i.e., available or not available). Abbreviations: IGME, Inter-agency Group for Child Mortality Estimation; OECD, Organization for Economic Co-operation and Development; UN SDG, United Nations Sustainable Development Goals; WHO, World Health Organization

Across country-level, web-based health inequality repositories, the types of health indicators and determinants of health reported varied considerably as did the years of coverage. Data were available from 1970 (Norway [28]) to 2022 (Canada [23]). Annual data were not always reported as in some instances, pooled estimates for three-to-six-year intervals were presented. The frequency at which data repositories were updated varied depending on the source of the data. Most data repositories did not have a consistent update schedule with some conducting multiple updates per year, since data were sourced from various institutions (Australia [21], World Bank [18], Norway [28], England [24], and Finland [27]) while others implemented annual updates (Eurostat [20], the WHO Health Inequality Data Repository [15], the US Diabetes Surveillance System [30], the UN SDG [17] and the IGME [19]). Some data repositories, such as Belgium [22], and Canada [23], timed updates to coincide with nationally administered surveys, or based on the availability of data from individual countries (the OECD [16]) leading to less frequent updates (ranging from every two to five years). Information on the frequency of updates was not always reported and in the case of the COVID-19 specific CHIME tool [26], no further updates were planned as of March 2023. Additionally, the availability of data on determinants of health varied within specific web-based health inequalities data repositories. For example, in Australia [21], health indicators stratified by sex were reported from 1982 onward while data stratified by indigenous status have been reported since 2008. Detailed characteristics of the web-based health inequalities data repositories identified are shown in Fig. 1.

### Health indicators (outcomes)

The number of distinct health indicators (outcomes) available from the identified web-based data repositories ranged from one (IGME [19]) to 139 (WHO [15]). On average, the identified web-based data repositories had 20 available health indicators.

**Fig. 2:**
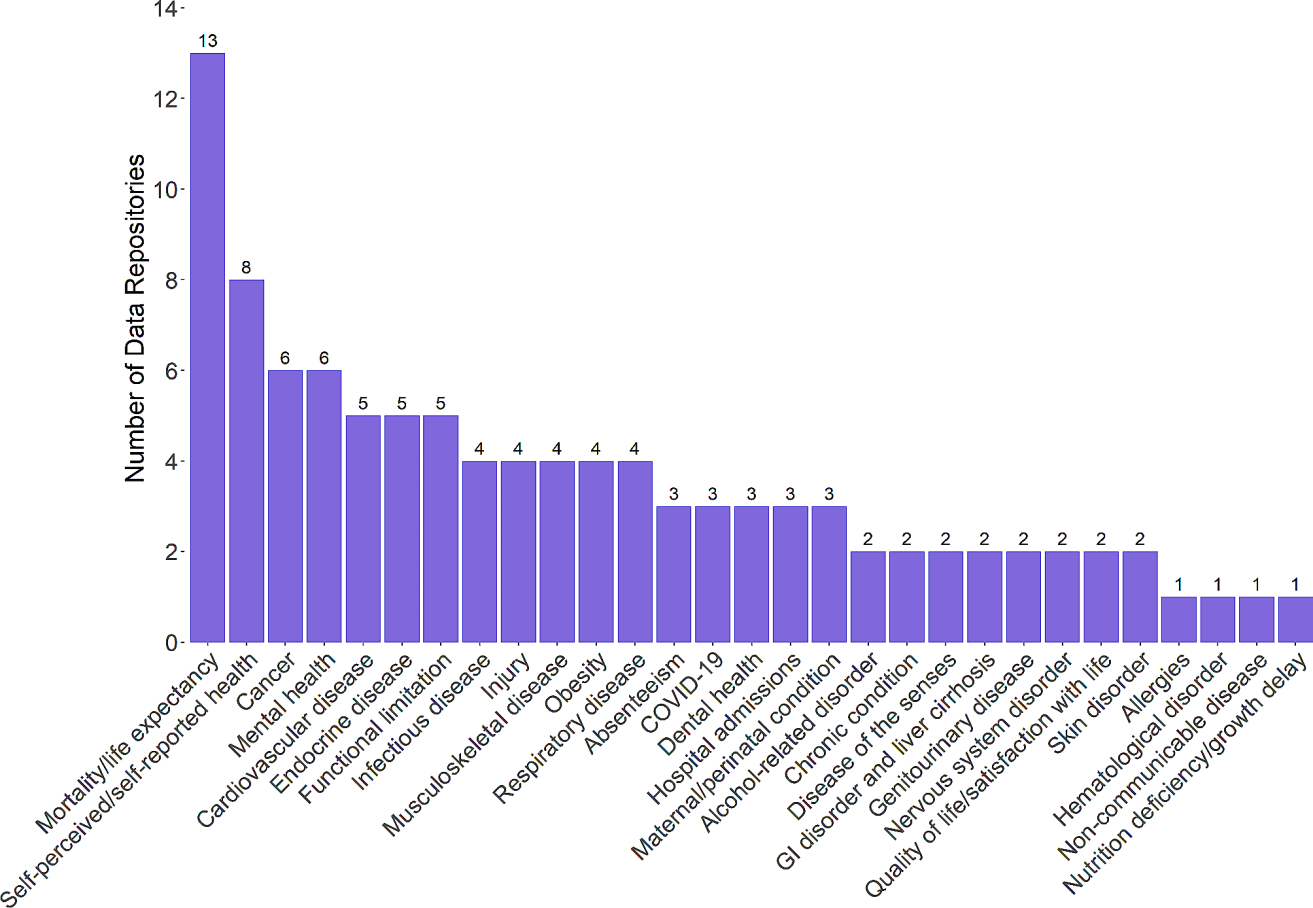
*Count of Data Repositories by Health Indicator Theme.* Abbreviations: COVID-19; coronavirus disease 2019; GI, gastrointestinal

Available health indicators were grouped by key themes as summarized in Fig. 2. Above and beyond mortality and life expectancy outcomes available from most data repositories (*n* = 13), the most commonly available health indicators were self-perceived/self-reported health (*n* = 8), cancer (*n* = 6), mental health (*n* = 6), cardiovascular disease (*n* = 5), endocrine disease including diabetes (*n* = 5), and functional limitation (*n* = 5).

Three data repositories (WHO [15], Australia [21], and England [26]) provided data relating specifically to the impact of COVID-19 on health indicators such as mortality rates, confirmed infection cases, and hospitalizations.

Mortality data were generally available for all-cause and disease-specific deaths. Infectious diseases, cardiovascular conditions, cancer, and respiratory diseases were the most frequently reported disease-specific causes of death. Few data repositories had data available for outcomes such as absenteeism (*n* = 3), hospitalizations (*n* = 3), and quality of life/satisfaction with life (*n* = 2) that typically inform cost-effectiveness analyses of new interventions. Data was available for absenteeism due to illness, but not for specific diseases or health conditions. The US [29] and the WHO [15] were the only two data repositories to provide disease-specific (other than due to COVID-19) hospitalization data. These included hospitalizations due to heart disease, stroke, chronic venous disease, asthma and chronic obstructive pulmonary disease, diabetes, and hip fractures. Quality of life and satisfaction with life were based on self-reported survey data.

### Determinants of Health (exposures)

**Fig. 3:**
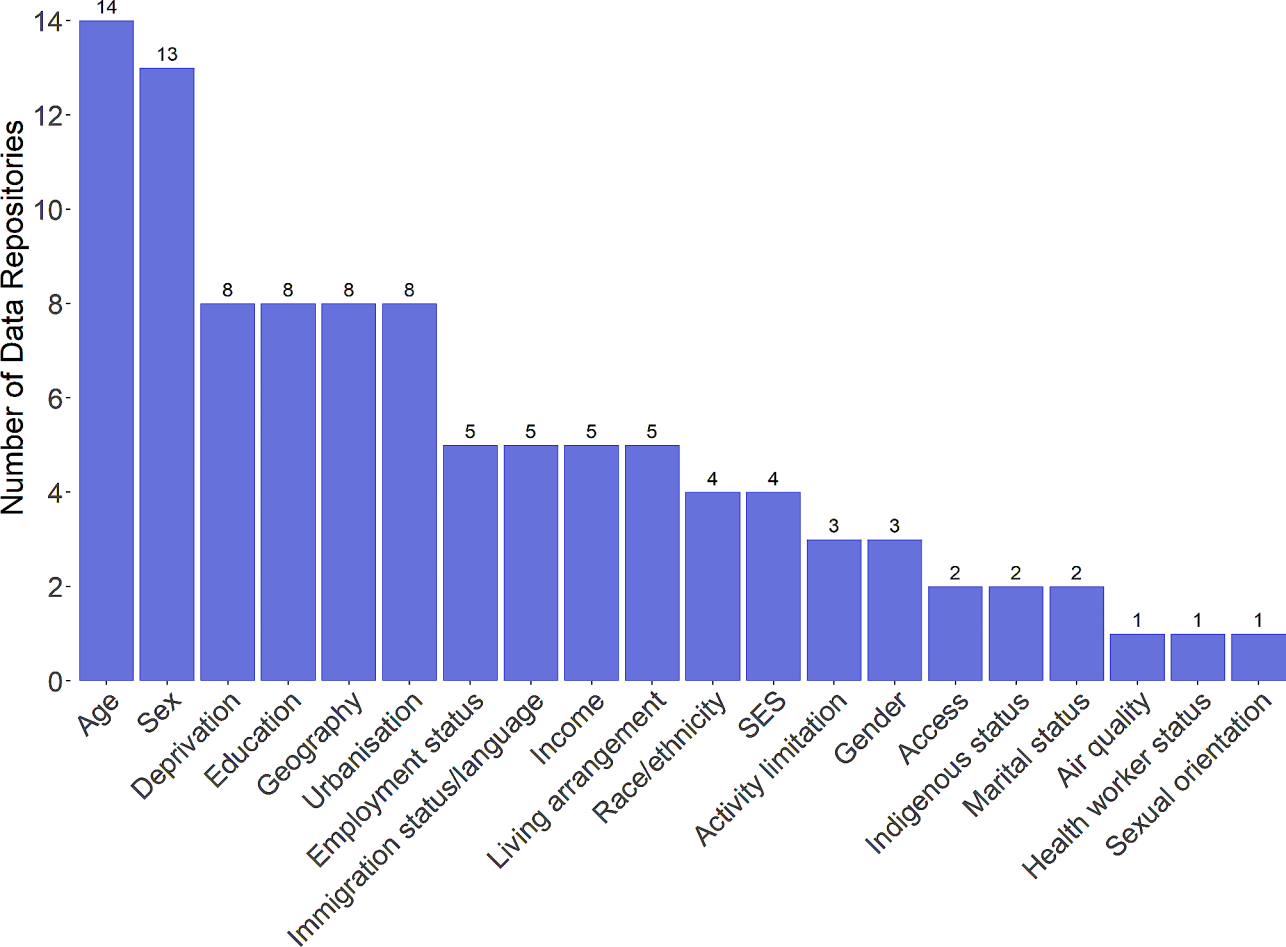
Count of Data Repositories by Determinants of Health. Abbreviation: SES, socioeconomic status

Twenty-one unique determinants of health (exposures) were identified across the included web-based data repositories, ranging from two (UN SDG [17], and World Bank [18]) to 24 (US [29, 30]). On average, the data repositories provided information on eight determinants of health. The most frequently available determinants of health themes were age (*n* = 14), sex (*n* = 13), deprivation (*n* = 8), education (*n* = 8), geography (*n* = 8), and urbanization (*n* = 8) (Fig. 3).

COVID-19-related data were available in three data repositories (WHO [15], Australia [21], and England [26]) across 13 determinants of health. Sex was reported in all three data repositories. Of note, the WHO [15] also included health worker status as a determinant of health of special significance in the context of COVID-19.

Three repositories (Belgium [22], Norway [28], and the US [29, 30]) reported on gender; however, no further information was provided on how these data were collected or defined. Canada [23], the US [29, 30], and England [24, 26] were the only countries to provide health indicator data stratified by race or ethnicity. Canada [23] was the only country to provide health indicator data stratified by sexual orientation. Australia [21] and Canada [23] provided health indicator data stratified by indigenous status.

The US [29, 30] reported considerably more determinants of health (at both the national and county levels) in comparison with the other data repositories including a number of unique measures around access to care and services (e.g., access to primary care, exercise opportunities, parks, food, and internet/computer).

**Fig. 4:**
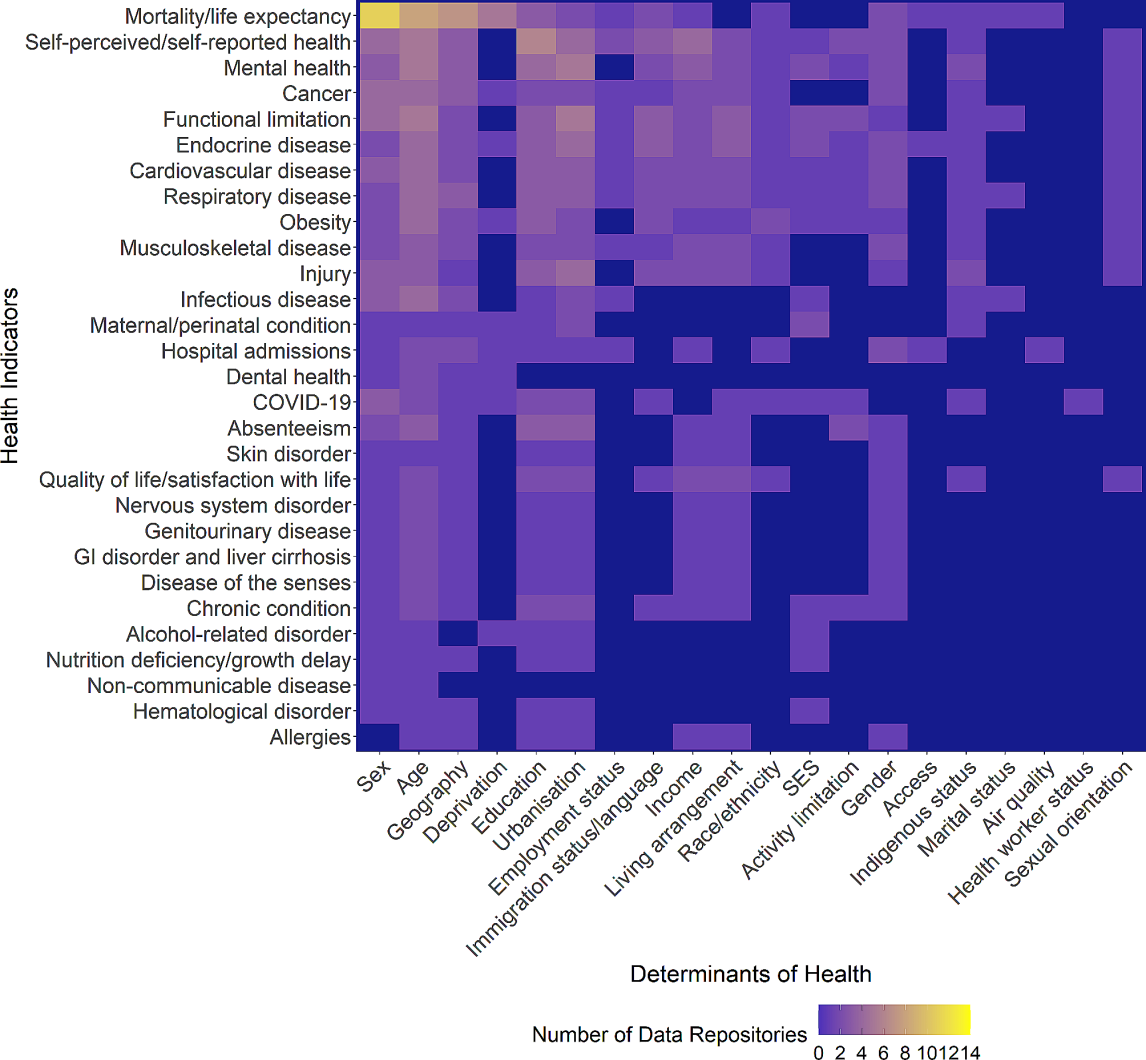
*Distribution of Health Indicators by Determinants of Health.* Abbreviations: COVID-19; coronavirus disease 2019; GI, gastrointestinal; SES, socioeconomic status

Within each data repository, disaggregated data were often not available for all health indicators or only for a subset of determinants of health. Figure 4 depicts the distribution of the number of data repositories providing disaggregated data for each pair of health indicator and determinant of health themes.

Mortality or life expectancy data disaggregated by sex (*n* = 11), age (*n* = 8), and geography (*n* = 7) were the most commonly available. Self-perceived/self-reported health, mental health, and functional limitation had data available in four data repositories or more for at least three determinant of health themes.

While only Canada [23] provided data disaggregated by sexual orientation, 11 different health indicator themes were covered. Similarly, only Canada [23], the US [29], and England [24, 26] reported disaggregated data by race/ethnicity, but 14 different health indicator themes were represented. England’s CHIME tool provided data on the impact of race/ethnicity on COVID-19 infections, hospitalizations, and deaths.

### Health Inequality Metrics

Only three data repositories (WHO [15], Canada [23], and England [24]) provided health inequality metrics. The WHO’s interactive user interface (the Health Equity Assessment Toolkit [HEAT]) enabled users to calculate ratio and difference summary measures of inequality. HEAT allowed users to explore inequalities in each setting of interest or to compare inequalities across selected settings.

England’s repository [24] reported on the relative index of inequality and the slope index of inequality, the two main indices used in epidemiologic research to quantify health inequalities within a population.

Canada’s repository [23] offered the highest number of metrics to quantify the magnitude of health inequalities between population subgroups. Effect measures (rate ratio, rate difference, and attributable fraction) were available to estimate the magnitude of inequality between two population groups. The availability of population impact measures (population-attributable rate, population-attributable fraction, and population impact number) also enabled users to estimate the impact of inequality between two population groups within the total population.

### Data repositories user features

All identified data repositories except for Belgium allowed users to download health indicator data disaggregated by the selected determinants of health. Comma-separated values and Microsoft Excel® (Redmond, WA, US) were the most frequently available data formats, with Finland [27] providing the largest variety of downloadable formats. The OECD [16] database also allowed users to download data in PC-Axis file format, which is a structured, text-based format designed for easy data dissemination and sharing, especially for statistical agencies.

Users were able to visualize data online in all identified data repositories except for Belgium. All remaining tools allowed visualization of data in table format; eight provided bar charts, five provided line graphs, and four provided maps or heatmaps. Eleven data repositories offered more than one type of data visualization. Most data visualizations did not include confidence intervals.

Most data repositories used drag-and-drop functions to first choose health indicator and add additional parameters (e.g., different determinants of health, years, output metrics). They also generally provided lists of data sources used and definitions for each variable included. The WHO [15] data were available through HEAT which is a software that provides an interactive platform where users can explore data, calculate health inequality summary measures, and generate unique visualizations.

## Discussion

The importance of measuring health inequalities and contextualizing health outcomes across different patient groups has been widely acknowledged in recent years as evidenced by the prominence of health equality considerations among the SDGs as well as the Rio Political Declaration on Social Determinants of Health [1, 2] and recent guidance by local HTA agencies (e.g., National Institute for Health and Care Excellence, Canadian Agency for Drugs and Technologies in Health). While providing accessible healthcare for all has been a key aim for most healthcare systems worldwide, there is evidence to suggest that specific, often marginalized, populations consistently experience poorer health outcomes due to a variety of socioeconomic and demographic differences [32–34]. A key aspect of the Rio Declaration was the commitment to develop robust data monitoring systems around health outcomes in order to unravel related inequalities [1]. While many countries publish annual progress reports on changes in health outcomes that consider various social determinants of health, the availability of disaggregated high-quality, comprehensive, and timely data remains limited [3].

Nationally and locally/regionally representative health-related data are regularly collected through well-established surveys (such as the Demographic and Health Surveys [35], and Multiple Indicator Cluster Surveys [36]). However, these sources can be difficult to navigate and are not publicly accessible. Against this background, an environmental scan was conducted to identify global and national, publicly accessible data repositories and summarize their key features.

Across identified data repositories, mortality and life expectancy were the most frequently reported health indicators, while age (closely followed by sex) was the most commonly reported determinant of health. The WHO Health Inequality Data Repository [15] emerged as the most comprehensive global data repository providing disaggregated data on various determinants of health. At the national level, data repositories were generally heterogeneous in terms of health indicators, determinants of health, health metrics, and years of coverage. Canada and England had the most complete national health inequality data repositories.

The identified data repositories and their interactive data exploration and visualization features may offer a unique opportunity for decision-makers to consider how to include health inequalities in assessing the value of new health technologies. Even though epidemiology and public health observatories have been monitoring trends in healthcare disparities for some time, including presenting evidence on the links between health outcomes and determinants of health, health technology regulation and market access (payer) decisions have not yet fully incorporated health equity as a distinct dimension of value when conducting HTAs [37–39]. Several previous publications have noted the lack of reliable and representative population-level health disparities data as one of the potential causes for neglecting health equity considerations in HTAs [5] and the important role of real-world evidence to shed light on these considerations. Furthermore, the collection and analysis of disaggregated data that accurately represent underserved and marginalized populations can be labor-intensive and time-consuming [39]. Publicly available data repositories may provide valuable insights during the different phases of the HTA process. During the HTA scoping phase, for example, they may help identify potentially marginalized groups and relevant determinants of health to consider in defining the decision problem [39]. Freely accessible data repositories may also provide the opportunity to assess how recommendations may generalize to specific population subgroups or how they may mitigate or exacerbate existing inequalities [39]. As health equity considerations are increasingly being incorporated in comparative effectiveness research and health economics modelling [8], publicly available data repositories may also provide reliable data (e.g., mortality, hospitalizations) to support these activities and help identify gaps that need to be addressed through primary data collection and standardization efforts. Trade-offs between health inequality data quality, biases, and the importance of locality in terms of health inequality data applicability (health inequality dimensions and unit of measurement) may affect decisions about data transportability across borders to unravel existing health disparities [40]. The choice of pertinent determinants of health for monitoring health inequalities should also align with the specific social conditions and policies of the local context, and this selection may vary between countries and settings and limit opportunities for data transportability [41].

The environmental scan revealed that, although publicly accessible data repositories offer potential for incorporating health inequalities into the HTA process, they are not without limitations. Disaggregated data were most commonly available for age, sex, geography, deprivation, and urbanization, but inequality patterns among other important dimensions (e.g., disability, race and ethnicity, sexual orientation) were very limited. The scarcity of data on drivers of health such as race or ethnicity and sexual orientation is a significant evidence gap as these factors are known to impact health outcomes and access to healthcare [42, 43]. Furthermore, despite ample reporting on sex and gender, the absence of clear definitions, lack of transparency on how these data were collected, and their reporting as binary variables could potentially mean that sex and gender were being used interchangeably and were therefore conflated [44–47]. There was also considerable variability in the years covered by data repositories and the frequency of data updates, which is an important factor when judging the relevance and quality of a particular source. Limited availability of health inequality summary measures may also restrict scenario analyses comparing two or more subgroups across health indicators, settings, and time [41, 48].

The environmental scan highlighted effective practices applicable to both existing and prospective data repositories. The consistent use of age, sex, social deprivation, education, geography, and urbanization determinants of health (exposures) allows for cross-national comparisons. However, additional determinants of health such as race/ethnicity and sexual orientation should be considered to address issues of intersectionality in addressing inequalities in these marginalized populations. Disease-specific health indicators (outcomes) such as mortality, resource use (e.g., hospitalizations), and quality of life could be used to derive equity weights for equity-informed economic evaluations. Health inequality metrics such as rate differences or ratio metrics are key to estimating the magnitude of inequality and would allow between-group comparisons. To monitor longitudinal changes in health inequalities, data repositories should be updated at regular intervals which are sufficiently long to observe changes and sufficiently short to ensure relevant data are available (e.g., every 2 to 5 years). Data visualization features and, more importantly, downloadable data formats should be provided to allow researchers to conduct health inequality analyses. Operational realities and population demographics should be factored in when establishing national health inequality repositories, recognizing country-specific variations in data accessibility and relevant health determinants.

The findings of this environmental scan should be considered within the context of some limitations. The search focused on data repositories that were available in English through web-based searches and no attempts were made to directly contact public health organizations to inquire about their databases. It is therefore possible that some potentially relevant databases were missed. Additionally, the search was focused on OECD countries as these have established healthcare information systems and the resources necessary for monitoring populations and health risks [3]. However, addressing health inequalities globally will require bridging the health inequality monitoring gap between high-income countries and low- to middle-income countries [49–51]. Further, it was beyond the scope of this environmental scan to appraise the quality of the data available from the identified data repositories in terms of their timeliness, completeness, accuracy, and reliability. Users of publicly available data repositories should take these aspects of data quality into account when utilizing or interpreting the data.

## Conclusions

This environmental scan offers an up-to-date overview of global and national publicly accessible web-based data repositories focusing on health inequalities, serving as a valuable tool for researchers and decision-makers involved in addressing disparities. While the WHO provides the most comprehensive global data repository, Canada and England stand out at the national level. However, the availability of disaggregated data is currently limited to a few determinants of health, emphasizing the need for broader representation to effectively monitor and address health inequalities. Nonetheless, promising practices were identified that should be considered when developing new global or national health inequality data repositories.

### Electronic supplementary material

Below is the link to the electronic supplementary material.

Supplementary Material 1

## Data Availability

No datasets were generated or analysed during the current study.

## Abbreviations

AHPF: Australian Health Performance Framework
CHIME: COVID-19 Health Inequalities Monitoring for England
COVID-19: Coronavirus disease 2019
HEAT: Health Equity Assessment Toolkit
HISIA: Belgian Health Interview Survey – Interactive Analysis
HTA: Health technology assessment
IGME: Inter-agency Group for Child Mortality Estimation
OECD: Organisation for Economic Co-operation and Development
SDG: Sustainable Development Goals
UN: United Nations
US: United States
WHO: World Health Organization
